# Oxidative stability of chilled broiler breast meat as affected by dietary supplementation with rosemary (*Rosmarinus officinalis* L.) powder and vitamin E

**DOI:** 10.1002/fsn3.474

**Published:** 2017-04-20

**Authors:** Hossein Rostami, Alireza Seidavi, Mohammad Dadashbeiki, Yadollah Asadpour, João Simões, Vito Laudadio, Chrysostomos Milis, Vincenzo Tufarelli

**Affiliations:** ^1^ Department of Animal Science, Rasht Branch Islamic Azad University Rasht Iran; ^2^ Department of Veterinary Science, Rasht Branch Islamic Azad University Rasht Iran; ^3^ Agricultural and Natural Resources Research Center of Guilan Rasht Iran; ^4^ Department of Veterinary Science University of Trás‐os‐Montes e Alto Douro Quinta de Prados Vila Real Portugal; ^5^ Department of DETO Section of Veterinary Science and Animal Production University of Bari ‘Aldo Moro’ Valenzano Bari Italy; ^6^ Ministry of Rural Development and Foods Feedstuffs’ Analysis and Control Laboratory Thessaloniki Greece

**Keywords:** antioxidants, breast meat, broiler, carcass quality, lipid oxidation

## Abstract

The aim of the present study was to evaluate the effect of rosemary (*Rosmarinus officinalis* L.) powder and vitamin E, as feed additives combined at different levels, on oxidative stability of broiler meat up to 14th day after chilling. A total of 270 1‐day‐old male chicks of Ross 308 strain were randomly assigned to nine dietary groups with three replicates having 10 birds each. Diets were supplemented with 0, 0.5, or 1.0% of rosemary (R) powder and 0, 100, or 200 mg/kg of vitamin E (alpha‐tocopherol acetate; VitE) according to the following treatments: T1 – control basal diet (0R + 0VitE); T2 – 0R + 100VitE; T3 – 0R + 200VitE; T4 – 0.5R + 0VitE; T5 – 0.5R + 100VitE; T6 – 0.5R + 200VitE; T7 – 1.0R + 0VitE; T8 – 1.0R + 100VitE; and T9 – 1.0R + 200VitE. At day 42, two birds of each replicate were slaughtered and the length and weight of cecum was recorded. Carcasses and their economically valuable parts were also weighted and broiler breast refrigerated at 4°C for 14 days. At day 0, 4, 7, and 14 of storage the malondialdehyde (MDA) content of breast meat was evaluated. After 4 days of storage, meat MDA contents of the T5 (0.43 mg/kg) and T9 (0.41 mg/kg) were lower than control group (T1: 0.55 mg/kg; *p* < .05). On day 7, groups supplemented with rosemary or VitE alone showed similar MDA contents (*p* > .05) than control. On day 14, lower (*p* < .05) MDA contents than T1 were observed in all groups except for broilers fed diet supplemented only with vitamin E. No effects were observed between treatments on the relative weight of the several carcass traits, however, VitE influenced (*p* < .05) the weight and size of cecum. Based on our findings, the combination of rosemary powder and vitamin E at different levels in diet is useful to limit the lipid oxidation of chilled chicken meat.

## 1 INTRODUCTION

In order to find effective substitutes to antibiotic growth promoters, several aromatic plants have been evaluated in poultry industry (Abudabos, Alyemni, Dafallah, & Khan, 2016; Khan, Naz, Nikousefat, Tufarelli, & Laudadio, 2012). These herbs, or their extract, show significant antimicrobial, antifungal, antioxidant, and physiological modulation proprieties (Ardalan, Omid, Golnaz, & Mahdi, 2013; Çabuk, Eratak, Alçicek, & Bozkurt, 2014; Jang, Ko, Kang, & Lee, 2007). Moreover, natural extracts also can serve as natural antioxidant limiting lipid oxidation in meat and meat products (Dhama et al., 2015; Shah, Bosco, & Mir, 2014; Tufarelli, Laudadio, & Casalino, 2016).

Rosemary (*Rosmarinus officinalis* L.) is a native plant of the Mediterranean countries widely used as aromatic and medicinal plant (Charles, 2013) having antioxidant proprieties (Carvalho, Moura, Rosa, & Meireles, 2005; Okoh, Sadimenko, & Afolayan, 2010; Yesilbag, Gezen, Biricik, & Meral, 2013). The high antioxidant activity was related with phenolic diterpenes (carnasol, rosmanol, 7‐methyl‐epirosmanol, isorosmanol, and carnosoic acid) and phenolic acids (rosmarinic and caffeic acids) (Alagawany & El‐Hack, 2015; Yesilbag, Eren, Agel, Kovanlikaya, & Balci, 2011). Some studies were conducted in order to evaluate the lipid oxidation of meat after refrigerated storage in broiler chicken fed diet supplemented with rosemary (including volatile oils) and/or vitamin E (Loetscher, Kreuzer, & Messikommer, 2013; Lopez‐Bote, Gray, Goma, & Flegal, 1998; Yesilbag et al., 2011). The lipid oxidation, an oxidative rancidity related with higher polyunsaturated fatty acids, is one major cause of meat deterioration (Botsoglou, Florou‐Paneri, Christaki, Fletouris, & Spais, 2002; Laudadio & Tufarelli, 2011; Morrisey, Brandon, Buckley, Sheey, & Frigg, 1997).

Despite the broiler diet with rosemary supplementation promotes oxidative stability of meat up to 9 days after chilling (Loetscher et al., 2013), at our knowledge, the effect of concomitant use of rosemary and vitamin E in diet at different levels on meat lipid oxidation was not yet reported. Moreover, the cecum development is important part of gut related with poultry growth performance (Mountzouris et al., 2010), and it is an economically relevant carcass characteristics and meat cuts remain poorly characterized when these natural additives are used in broiler diets. Therefore, the objective of this study was to determine the effect of different dietary levels of rosemary powder and vitamin E to broilers on the oxidative stability of breast meat up to 14 days after chilling at 4°C and to characterize the economically valuable carcass traits when both natural additives were supplemented.

## 2 MATERIAL AND METHOD

### 2.1 Animals and housing

This experiment was performed at the poultry house of the Agricultural and Natural Resources Research Center of Guilan, Guilan, Rasht, Iran, and also at the Faculty of Agriculture, Islamic Azad University, Rasht Branch, Rasht, Iran. All procedures were approved by the Authors’ Institution Ethic Committee.

In order to allocate a total of 270 one‐day‐old male chicks of the Ross 308 strain (Aviagen, Newbridge, Scotland, UK) for 42 days, a 5 × 20 m facility with six ventilators was used. The temperature program followed the manual instructions for Ross 308 breeding (Aviagen, Newbridge, Scotland, UK) and the air relative humidity varied from 55% to 65%. Twenty‐one hours lighting was on and daily for 2 hr between 19:00 and 22:00 the house was left dark till slaughter at 42nd day. Prior to the experiment the facility was carefully cleaned including all drinkers and feeders which were washed daily during whole experiment duration.

### 2.2 Study design and diet composition

The experimental design included nine treatments with three replicates for each dietary treatment. Each replicate had 10 birds, such that mean body weights were similar between groups. All chickens were fed according to the producer's feeding instructions. The composition of basal diet and its nutrient composition in the starter (1–21 days of age) and finisher (22–42 days of age) rearing periods are given in Table 1.

**Table 1:** Ingredient and chemical composition of the basal diet fed to broiler chickens

Ingredient (g/kg as fed)	Starter	Finisher
Corn	556.3	585.9
Soybean meal	355.3	322.3
Soybean oil	27.4	43.7
Gluten meal	10.0	—
Wheat bran	7.0	10.0
CaCO_3_	12.9	10.5
Ca_%22_P_%18_	18.9	16.7
NaCl	2.3	2.7
Mineral mixture^a^	3.0	3.0
Vitamin mixture^b^	3.0	3.0
NaHCO_3_	1.9	1.2
DL‐Methionine	1.2	1.0
Lysine‐Hydro‐Chloride	0.8	—
Nutrients (%, calculated values)		
Dry matter	86.5	87.1
Metabolizable energy (MJ/kg)	12.7	13.2
Crude protein	230	210
Ether extract	53.0	70.0
Linoleic acid	28.0	37.0
Crude fiber	27.0	27.0
Calcium	10.5	9.0
Phosphorus	7.4	6.9
Available phosphorus	5.0	4.5
Potassium	9.0	8.4
Sodium	1.6	1.6
Glycine	9.3	8.6
Lysine	12.7	11.1
Methionine	4.7	4.2
Met + Cys	8.5	7.6
Tyrosine	8.9	8.1
Threonine	8.3	7.8
Tryptophan	3.0	2.8
Arginine	13.0	13.0

a Cu: 3 mg/g; Zn: 15 mg/g; Mn: 20 mg/g; Fe: 10 mg/g; K: 0.3 mg/g.

b Vitamin A (retinyl acetate) 3 mg/kg; vitamin D_3_ (cholecalciferol) 0.12 mg/kg; vitamin E (DL‐alpha‐tocopheryl acetate) 3 mg/g; vitamin K_3_ (2‐methyl‐1,4‐naphthoquinone) 1.5 mg/g; vitamin B_6_ (pyridoxines) 13 mg/g; riboflavin 1 mg/g; calcium pantothenate 4 mg/g; niacin 15 mg/g.

The basal diet was supplemented with 0, 0.5, or 1.0% of rosemary (R) powder and 0, 100, or 200 mg/kg of vitamin E (alpha‐tocopherol acetate; VitE**)**, according to each treatment: T1 – control basal diet (0R + 0VitE); T2 – 0R + 100VitE; T3 – 0R + 200VitE; T4 – 0.5R + 0VitE; T5 – 0.5R + 100VitE; T6 – 0.5R + 200VitE; T7 – 1.0R + 0VitE; T8 – 1.0R + 100VitE, and T9 – 1.0R + 200VitE. Rosemary powder had the following chemical composition: gross energy 13.1 MJ/kg; metabolizable energy 10.5 MJ/kg; crude protein 436.0 g/kg; crude fiber 207.3 g/kg; based on AOAC method (AOAC, 2005) and powder was added by mixing to the basal diet of respective groups.

### 2.3 Carcass processing procedures

At the day 42, two birds from each replicate were slaughtered, after 4 hr of fasting, to measure the cecum characteristics, carcass yield, and meat cuts. Dry pecking method was used. Feet, neck, wingtips, gut, and liver were removed and the edible carcass was weighed. The right and left cecum were washed, weighed, and their width, length, and diameter measured. Each carcass was dissected and economically relevant meat cuts were weighed: breast muscle including skin and sternum, legs (thighs and drumsticks), and wings. The breast from each carcass was refrigerated at 4°C for 14 days and assessed for oxidative stability on the day of slaughter (day 0) and after 4, 7, and 14 days of storage.

### 2.4 Meat oxidation evaluation

The Thiobarbituric acid (TBA) was determined based on the description of Tarladgis, Watts, and Yonathan (1960). This tissue TBA assay is considered as a standard method for malondialdehyde (MDA) analysis (Tokur, Korkmaz, & Ayas, 2006) and involved the reaction between two molecules TBA and one molecule MDA. Each homogenized sample was added to 97.5 ml of distilled water and 2.5 ml of 6 N HCl and distilled until reaching 200 ml of distillate. A total of 5 ml of thiobarbituric reactive reagent (0.02 M TBA in 90% glacial acetic acid) was added in equal part to 5 ml of distillate. This mixture was incubated for 35 min on boiling water. After cooling, the absorbance was measured at 538 nm. The multiplication by 7.8 was used in order to calculate the distillation TBA number as described by Tarladgis et al. (1960).

### 2.5 Statistical analysis

The following formula: [(weight of component(s)/eviscerated carcass weight) × 100] was used for respective ratios calculation. Data were tested by analysis of variance using a 3 × 3 factorial design with three rosemary (0, 0.5, and 1.0%) and three vitamin E (0, 100, and 20 mg/kg in diet) levels, using the two‐way ANOVA procedure. Data were analyzed using SPSS (1997) statistical software and the GLM procedure was used. The means (±SEM) were compared by using least significant difference (LSD). Results were considered significantly different at *p* < .05.

## 3 RESULTS

A significant effect (*p* < .05) of both rosemary powder and vitamin E dietary supplementation on MDA contents of breast meat broiler was observed on 4th, 7th, and 14th day after chilled storage (Table 2). In overall, MDA contents were lower in groups of broilers supplemented with rosemary powder plus vitamin E.

**Table 2:** Thiobarbituric acid (TBA) value presented as malondialdehyde (MDA) content (±SEM) of breast muscle stored at 4°C for 14 days in broilers fed diets containing different levels of rosemary powder and vitamin E

Treatment	Day of storage (mg MDA/kg of muscle)			
	0	4	7	14
Rosemary (%)				
0	0.36	0.50^a^	0.63^a^	0.74^a^
0.5	0.35 (±0.01)	0.43^b^ (±0.01)	0.59^b^ (±0.01)	0.63^b^ (±0.02)
1.0	0.34	0.47^ab^	0.62^ab^	0.64^b^
Vitamin E (mg/kg)				
0	0.36	0.50^a^	0.63^a^	0.71^a^
100	0.35 (±0.01)	0.47^ab^ (±0.01)	0.61^ab^ (±0.01)	0.67^ab^ (±0.02)
200	0.34	0.42^b^	0.60^b^	0.63^b^
T1 (0% + 0 mg/kg)	0.38	0.55^a^	0.67^a^	0.82^a^
T2 (0% + 100 mg/kg)	0.35	0.50^ab^	0.60^ab^	0.73^ab^
T3 (0% + 200 mg/kg)	0.35	0.44^ab^	0.61^ab^	0.68^ab^
T4 (0.5% + 0 mg/kg)	0.36	0.45^ab^	0.60^ab^	0.65^b^
T5 (0.5% + 100 mg/kg)	0.36 (±0.01)	0.43^b^ (±0.02)	0.59^b^ (±0.01)	0.63^b^ (±0.03)
T6 (0.5% + 200 mg/kg)	0.32	0.42^b^	0.60^b^	0.60^b^
T7 (1.0% + 0 mg/kg)	0.34	0.49^ab^	0.63^ab^	0.66^b^
T8 (1.0% + 100 mg/kg)	0.35	0.49^ab^	0.63^ab^	0.64^b^
T9 (1.0% + 200 mg/kg)	0.34	0.41^b^	0.59^b^	0.62^b^

Different letters within the same column indicate significant differences among treatment groups (*p* < .05).

Rosemary powder influenced (*p* < .05) the weight and relative weight of the right cecum. An effect of vitamin E was observed on length of right and left cecum (*p* < .05; Table 3). However, no significant differences (*p* > .05) were found between dietary groups for the right cecum width (8.09 mm) and diameter (0.36 mm). The left cecum weigh, relative weight, width, and diameter were as follows: 6.77 g, 0.28%, 8.36 mm, and 0.36 mm, respectively, without significant differences (*p* > .05) between groups.

**Table 3:** Mean (±SEM) of cecum at 42nd day of age in Ross 308 broilers fed diets containing the different levels of rosemary powder and Vitamin E

Treatment	Trait			
	Right cecum weight (g)	Relative weight of right cecum (%)	Right cecum length (mm)	Left cecum length (mm)
Rosemary (%)				
0	6.45^ab^	0.263^a^	182.22	190.00
0.5	8.31^a^ (±0.54)	0.336^b^ (±0.02)	192.22 (±4.80)	195.56 (±4.54)
1.0	6.25^b^	0.269^ab^	181.11	180.56
Vitamin E (mg/kg)				
0	6.93	0.29	192.78^a^	196.11^a^
100	7.65 (±0.54)	0.31 (±0.02)	189.44^ab^ (±4.80)	197.22^a^ (±4.54)
200	6.43	0.27	173.33^b^	172.78^b^
T1: (0% + 0 mg/kg)	6.13	0.25^ab^	188.33	191.67^ab^
T2: (0% + 100 mg/kg)	7.87	0.33^ab^	191.67	196.67^ab^
T3: (0% + 200 mg/kg)	5.35	0.21^a^	166.67	181.67^ab^
T4: (0.5% + 0 mg/kg)	9.42	0.39^b^	203.33	206.67^a^
T5: (0.5% + 100 mg/kg)	6.80 (±0.93)	0.26^ab^ (0.03)	191.67 (±8.32)	206.67^a^ (±7.86)
T6: (0.5% + 200 mg/kg)	8.69	0.36^ab^	181.67	173.33^ab^
T7: (1.0% + 0 mg/kg)	5.24	0.23^ab^	186.67	190.00^ab^
T8: (1.0% + 100 mg/kg)	8.28	0.35^ab^	185.00	188.33^ab^
T9: (1.0% + 200 mg/kg)	5.23	0.24^ab^	171.67	163.33^b^

Different letters within the same column indicate significant differences among treatment groups (*p* < .05).

No significant differences (*p* > .05) among treatments on broilers’ live body weight and empty abdomen carcass weight as well as eviscerated carcass percentage (Table 4) or on breast and drumsticks relative weight were observed (Table 5).

**Table 4:** Economically relevant carcass characteristics in broilers fed diets containing different levels of rosemary powder and vitamin E

Treatment	Trait				
	Live body weight (g)	Defeather body weight (g)	Full abdomen carcass weight (g)	Empty abdomen carcass weight (g)	Eviscerated carcass (%)
Rosemary (%)					
0	2813.9	2473.7	2305.6	1767.8	76.6
0.5	2785.0 (±100.5)	2467.8 (±80.2)	2278.9 (±76.4)	1795.0 (±63.3)	78.8 (±0.7)
1.0	2632.2	2328.9	2140.0	1683.9	78.7
Vitamin E (mg/kg)					
0	2715.0	2393.7	2207.2	1696.1	76.7
100	2763.9 (±100.5)	2457.8 (±80.2)	2260.6 (±76.4)	1781.1 (±63.3)	78.8 (±0.7)
200	2752.2	2418.9	2256.7	1769.4	78.6
T1: (0% + 0 mg/kg)	2785.1	2431.2	2253.3	1696.7	75.1
T2: (0% + 100 mg/kg)	2746.7	2418.4	2221.7	1735.0	78.1
T3: (0% +200 mg/kg)	2910.1	2571.7	2441.6	1871.6	76.7
T4: (0.5% + 0 mg/kg)	2770.2	2423.3	2226.7	1750.1	78.5
T5: (0.5% + 100 mg/kg)	2830.0 (±174.0)	2573.3 (±138.9)	2373.3 (±132.3)	1865.0 (±109.6)	78.6 (±1.2)
T6: (0.5% + 200 mg/kg)	2755.0	2406.8	2236.8	1770.2	79.4
T7: (1.0% + 0 mg/kg)	2590.2	2326.6	2141.7	1641.7	76.6
T8: (1.0% + 100 mg/kg)	2715.0	2381.7	2186.5	1743.3	79.7
T9: (1.0% + 200 mg/kg)	2591.7	2278.3	2091.7	1666.8	79.7

**Table 5:** Economically valuable meat cuts in relation to eviscerated carcass in broilers fed diets containing the different levels of rosemary powder and vitamin E

Treatment	Trait			
	Breast weight (g)	Relative breast weight (%)	Drumsticks weight (g)	Relative drumsticks weight (%)
Rosemary (%)				
0	845.5	34.1	716.7	28.9
0.5	845.2 (±31.8)	34.3 (±0.5)	722.9 (±31.0)	29.3 (±0.5)
1.0	789.2	33.9	673.0	28.9
Vitamin E (mg/kg)				
0	809.7	33.8	688.7	28.7
100	826.2 (±31.8)	33.6 (±0.5)	719.8 (±31.0)	29.3 (±0.5)
200	844.0	34.9	704.0	29.1
T1: (0% + 0 mg/kg)	820.6	33.7	709.0	28.9
T2: (0% + 100 mg/kg)	802.7	33.2	696.9	28.8
T3: (0% + 200 mg/kg)	913.2	35.5	744.1	28.9
T4: (0.5% + 0 mg/kg)	837.5	34.5	703.4	29.0
T5: (0.5% + 100 mg/kg)	862.3 (±55.1)	33.5 (±0.9)	763.2 (±53.8)	29.6 (±0.9)
T6: (0.5% + 200 mg/kg)	835.9	34.8	702.0	29.2
T7: (1.0% + 0 mg/kg)	771.2	33.1	653.7	28.1
T8: (1.0% + 100 mg/kg)	813.6	34.1	699.2	29.4
T9: (1.0% + 200 mg/kg)	782.9	34.4	666.1	29.2

## 4 DISCUSSION

Thiobarbituric acid (TBA) analysis is an indicator of malondialdehyde (MDA), a product of oxidation that increases during the storage period (Yesilbag et al., 2011). The results of our study showed that the rosemary supplementation in broiler diet combined with vitamin E, but not as single additive, was effective in order to prevent the breast meat lipid oxidation, decreasing the MDA contents when compared with the control group during the different days of storage. Yesilbag et al. (2011) observed a decrease in MDA concentrations on 1st, 3rd, and 5th day after meat storage when broilers were fed with rosemary powder (5.7, 8.6, or 11.5 g/kg) or rosemary essential oil (100, 150, or 200 mg/kg) in comparison with broilers fed with 50 mg (control group) or 200 mg/kg of alpha‐tocopherol acetate. Although, these researchers reported the lack of significant differences between levels of each additive when linear and quadratic contrasts analysis was used, they concluded that rosemary plant could decrease lipid oxidation of broiler meat. However, an effect of dietary alpha‐tocopherol acetate supplement (200 mg/kg) on retarded lipid oxidation of meat enriched with n3‐polyunsaturated fatty acids was observed by Basmacioğlu, Tokuşoğlu, and Ergül (2004). These Authors also observed an higher alpha‐tocopherol acetate level after 15 days of meat refrigeration when 300 mg/kg of rosemary essential oil was added to diet, suggesting a protective role of this natural additive. Recently, Loetscher et al. (2013) observed a decrease in lipid oxidation and elevated tocopherol content of breast meat at day 9 of storage when broiler diet was supplemented with 2.5% of rosemary. A dietary supplement effect with 150 mg/kg or 300 mg/kg of rosemary essential oil on breast and thigh meat MDA contents was observed on 15th day after storage when compared with nonsupplemented broiler diets by Basmacioğlu et al. (2004). In our study, after 14 days of storage, dietary supplementation with rosemary alone at both levels (100 or 200 mg/kg) (T4 – 0.65 mg/kg and T7 – 0.65 mg/kg) showed lower meat MDA contents than in control group (0.82 mg/kg; *p* < .05). Contrarily, both single vitamin E supplementations (T2: 0.73 mg/kg and T3: 0.68 mg/kg) had similar (*p* > .05) MDA contents compared to control group. This result may support the hypothesis that the alfa‐tocopherol protection on meat is complemented by rosemary supplementation in broilers diet, such as also previously reported by Basmacioğlu et al. (2004).

In the present study, the weight and relative weight of carcass and several carcass parts were similar among treatments, suggesting that the 0.5 and 1.0% of rosemary supplementation with or without vitamin E have no adverse effect on carcass biometric characteristics. However, the increment of weight and relative weight of right cecum, when 0.5% rosemary powder was added or the small length of cecum when 200 mg/kg vitamin E was added, suggested a real influence of both additive in the organ development of broilers. In fact, the feeding rosemary can improve the body weight gain, but not the final live weight of broilers, as reported by Yesilbag et al. (2011). However, Basmacioğlu et al. (2004) observed a decrease in weight gain at the end of the starter feed period (21st day) in broilers fed diet including rosemary. This increment was not always evident, and Yesilbag et al. (2011) also observed no significant differences with rosemary supplementation on broilers live weight gain.

In conclusion, supplementing rosemary and vitamin E in diet at 0.5% + 100 mg/kg and 1.0% + 200 mg/kg, respectively, reduced significantly the lipid oxidation of chilled chicken meat at 4th, 7th, and 14th day without any adverse effect on the carcass parts and meat cuts.

## CONFLICT OF INTEREST

None declared.
